# To schedule or not to schedule? Agentic and cooperative teams at call centers

**DOI:** 10.3389/fpsyg.2014.00999

**Published:** 2014-09-16

**Authors:** Danilo Garcia, Erik Lindskär, Trevor Archer

**Affiliations:** ^1^Network for Empowerment and Well-BeingGothenburg, Sweden; ^2^Centre for Ethics, Law and Mental Health, Institute for Neuroscience and Physiology, University of GothenburgGothenburg, Sweden; ^3^Department of Psychology, University of GothenburgGothenburg, Sweden

**Keywords:** agency, communion, call center, self-managing teams, responsibility, cooperation, self-directedness, well-being

Work at call centers is often designed around technical solutions that imply some type of work schedule—every second that an agent is not on the phone amounts to precious queue time that must be managed (Durrande-Moreau, 1999). Even activities such as coffee brakes are scheduled (Garcia et al., 2012) and most call centers define a minimum percentage of the scheduled “time on the phone” (Garcia and Archer, 2012). This type of work design might imply unfavorable working conditions for employees, which in turn affect well-being, learning, and how agents cope with the rapid external and internal changes in working life. Indeed, performance at call centers (measured as the percentage of time on the phone/scheduled phone-time) has been shown to be negatively related to important work climate aspects (e.g., sense of autonomy and responsibility, relation with managers and colleagues; Garcia and Archer, 2012), employees' view of how successful the organization is in reaching its core values (e.g., communal values such as helpfulness toward the customer or colleagues; Garcia and Archer, 2012), and also employees' well-being (e.g., positive affect, life satisfaction). Scheduling agents' time on the phone might also limit their ability to work efficiently within the allocated working time (i.e., performance), probably because the amounts of incoming calls are completely outside the leaders' or employees' control—a common characteristic of workplaces in which services are delivered by phone (Ryan and Ployhart, 2003). A work situation with high demands and low freedom, through rigorous control of working procedures, creates a feeling of lack of control which can cause mental overload, in turn, leading to mental and physical health problems. Moreover, the low level of responsibility that is also common in call centers (e.g., employees do not need or are not expected to make decisions to improve services), along the lack of environmental control and performance monitoring, might influence agents to become passive (Karasek, 1979) and disempowered (Archer et al., 2014; Jimmefors et al., 2014).

Recently, together with our colleagues we have also found that individuals' communal character traits (i.e., the tendency to care and help others and being tolerant and empathic) are negatively associated to performance at call centers over a 6-month period. In other words, call centers seem to indeed disempower workers by scheduling every single task and by individualizing the way performance is measured, which diminishes their sense of autonomy and responsibility (i.e., agency or Self-directedness) and helpful behavior, social tolerance and empathy (i.e., communion or Cooperativeness). This is extremely counterproductive; especially in light of what call centers' agents state is the most positive factor in their work environment: their colleagues. Figure 1, for instance, shows a word cloud of the most common used words by 368 call center employees (unpublished data retrieved from Garcia and Archer, 2012) when describing positive things with their workplace (the size of the words corresponds to how often the word co-occurs in the text generated by the agents). In contrast to this notion, our results showed that call center agents with high levels of self-control and low levels of communal values are the ones performing the highest in this work environment. In most recruitment situations the main focus is to match the individual to the task or work environment (e.g., Garcia et al., 2014). Although this recruitment practice is somewhat appropriate, when applied in the recruitment of call center agents it might lead to recruitment of personnel high in self-control and low in cooperation. In other words leaving out workers that are high in agentic and communal core values (i.e., responsibility and cooperation).

**Figure 1:**
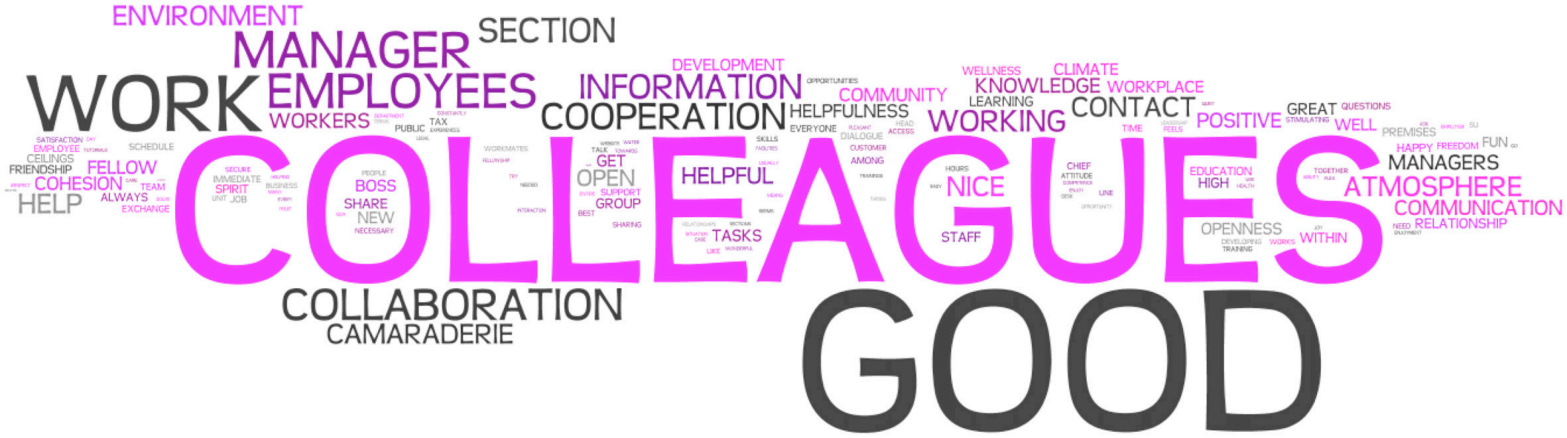
**Call center agents' (*N* = 368; see Garcia and Archer, 2012) statements of the most positive with their work depicted as a word cloud (created with www.wordle.net)**. Note: The words were translated from Swedish to English. The font size is proportional to the frequency of the word, the most frequent the word is in the statements the bigger font size.

Moreover, when groups and goals are shaped in an organization, it is easy for managers and employees to think “input-output models,” for example, assume that homogeneity (i.e., all members of the group have similar characteristics or competencies) leads to higher performance (Wageman, 2001). Both leaders and co-workers think that other co-workers who are perceived as being of one's own “kind” raise the groups' competence (Wageman, 2001). However, having an extremely skilled team in which individuals are as capable or even overqualified for the task will not lead to any improvement in performance (Wageman, 2001).

In this opinion article, we propose the concept of self-managing teams (Hackman, 1987, 1990) as an alternative work design for call centers. As is common for work teams, the structure and purpose of a self-managing team is decided by others (e.g., customers, leaders), however, self-managing teams have the authority and accountability for not only executing the task but also monitoring and managing work processes—initiating changes in pace or procedure as needed. It is our opinion that such work design leads to the internalization and/or exploitation of agentic and communal values that positively influence workers' well-being and performance, thus, empowering the individual and the organization. Empowerment implies the capacity for self-awareness and knowledge together with the power and strength to take responsibility (Garcia et al., in review); these attributes are associated with the ability to make the right decisions regarding different aspects of one's and others' well-being (Garcia et al., in review). Well-being in this context refers to feeling good (i.e., happiness), doing good (i.e., mature and actively virtuous living), physical health (i.e., absence of disease or infirmity), and prosperity (i.e., success, good fortune, and flourishing), see Cloninger (2004, 2013).

In general, in a goal-oriented organization, the following “must” work: (1) the individual or group must actually perform the work, (2) the individual or group must monitor and take care of the work and if necessary also do change in work rate or strategy, (3) the individual or group must organize the group and its environment (e.g., by assigning the task, arranging support and resources within the organization) and finally, (4) an individual or group must specify the objectives to be achieved (Hackman, 1987). A self-managing group has the authority and responsibility for the first two “must” (i.e., carry out the work, monitor/follow-up and take care of the work), but within certain parameters and objectives decided by others. Consequently, a group's level of empowerment in this two “must” is what defines it as a self-managed group or not.

The notion of self-managing teams might be useful because research on how leaders and groups interact shows that the group's performance is affected by three factors: (a) the degree of effort members of the group spend together to perform the work, (b) how well suited the group's performance strategy is to the task, and (c) the amount of knowledge and skills that members bring with them to carry out the task (Wageman, 2001). In practice this means that the level of limitation a work group has, in these three factors, influences different mechanisms at group and individual levels, which either lowers or raises the group's performance. The leader's ability to influence the group's effort will in turn be related to his or her ability to control over what circumstances might limit these three performance factors (Hackman and Wageman, 2005). In other words, a leader who cannot or do not engage in these factors will fail in her/his attempts to improve group performance. The more influence a group or leader has on these factors should affect work performance because the individual might experiencing a higher degree of control and responsibility over her/his own work and also higher trust in others. The number of incoming calls is, however, a huge restriction on the group and leaders of a call center, because the calls are largely beyond their control. Also, the competence of the group is limited by the task it self. Many call centers have simple tasks or personnel who are overqualified; both of these conditions limit either agents' opportunities to develop or has little influence on agents' performance (e.g., Swaab et al., 2014). Having opportunities to develop is, for instance, a predictor of well-being (i.e., positive and negative affect, life satisfaction, psychological well-being) and performance (i.e., percentage of time on the phone) among call center agents. Hence, it may be more effective to focus on the conditions that make it easier for groups to find the most appropriate strategy to solve the task. That is, making them self-managing.

Of course there are circumstances in which the group can influence the individual to perform more. Besides the size of the group (4–7 members is optimal according to Wageman, 1995), it is important that a self-managing team (I) has a clear direction and a clear objective of what should be done but not how, (II) has an optimal variety of skills relevant to the objective, (III) has an objective that allows or influence members to work together to manage it, (IV) has objective performance goals that requires effort, exceeding previous goals with specific deadlines and feedback if the goal has been reached or not, and (V) sets up strategic explicit norms or rules by convening meetings to solve problems, taking initiative for change in work habits, experimenting with new approaches, taking in good habits practiced by other groups, initiating solutions to problems, and discussing individual responsibility and contribution.

Furthermore, the notion of self-managing teams involves agentic and communal values, thus, it might have repercussions for employees' well-being. This is important not only from a mental health perspective but also from a business perspective—employees who feel good or are happy (i.e., experiencing positive emotions more often than negative emotions in their workplace) infect their mood to others, such as colleagues and customers (Ryan and Ployhart, 2003). For instance, happy employees engage more often in pro-social behavior determined by the employer, such as sharing their knowledge with colleagues. The happy employee is more willing to help colleagues and customers beyond the employer's expectations (i.e., “going the extra mile,” George, 1990). In other words, the implementation of self-managing teams as a work design practice in call centers might increase well-being, responsibility, and cooperation. The increasing of positive emotions among employees, in turn, reinforcing cooperation among team members and agents' helpful behavior toward customers; which increases productivity (Tjosvold et al., 2014). Nevertheless, it ought to be said that the link between happy employees/high performance is ambiguous. While public and private sector employees' positive emotions are related to work performance (Zelenski et al., 2008), this seems not to be the case among call center agents. Instead, it seems like thinking about their performance primes positive emotions among call center agents (Garcia et al., in press).

There are some indications from empirical research from Xerox Corporation's customer service department, various airline crews and IBM's programming team, suggesting that self-managing teams might have a place in the call center environment (for a review see Hackman and Wageman, 2005). Some of these organizations, although having similar purposes, have even more complex structure than most call centers. Xerox Customer Service Department, for example, is split into nine geographical areas, which in turn have subdivisions. Each subdivision is in turn composed of 5–10 teams in different cities, organized after the geography or the type of machines to be serviced. A team's main tasks are to answer customer calls about engine failure and to perform site visits for hardware maintenance. The research conducted at Xerox's customer services department, shows that self-managed teams are more effective than control groups (see Hackman and Wageman, 2005). Nevertheless, most call centers still employ the conventional work design detailed at the beginning of this Opinion article.

Whether it's a social movement (e.g., Martin Luther King's struggle against racism in the US) or business success (e.g., Ingvar Kamprad's IKEA), the leader is always seen as the key factor that affects individuals' willingness to perform beyond the ordinary. The leader's ability to communicate the organization's vision, purpose and goals has been shown to have an impact on employees' level of stress (Den Hartog and Koopman, 2002). A leader's achievement is often attributed to their personality or even innate characteristics, rather than the circumstances or the nature of the strategic choices they make or choose (see for a critical review Bligh and Schyns, 2007). Although this is important, in this opinion article we have not focused on how much a leader can influence workers' performance. Instead, we have defined what conditions the leader should create for call center groups to work as self-managing groups and not merely as a set of individuals (see also Luria, 2008). More importantly, this notion might empower the individual, the team and the organization to feel good (i.e., happiness), do good (i.e., mature and actively virtuous living), physical health (i.e., absence of disease or infirmity), and prosperity (i.e., success, good fortune, and flourishing).

“I suppose leadership at one time meant muscles; but today it means getting along with people”

Mohandas Gandhi

## Conflict of interest statement

The Associate Editor Ann-Christine Andersson Arntén declares that, despite having collaborated with the authors Danilo Garcia and Trevor Archer the review process was handled objectively and no conflict of interest exists. The authors declare that the research was conducted in the absence of any commercial or financial relationships that could be construed as a potential conflict of interest.
